# Glutamine synthetase 2 is not essential for biosynthesis of compatible solutes in *Halobacillus halophilus*

**DOI:** 10.3389/fmicb.2014.00168

**Published:** 2014-04-14

**Authors:** Anna Shiyan, Melanie Thompson, Saskia Köcher, Michaela Tausendschön, Helena Santos, Inga Hänelt, Volker Müller

**Affiliations:** ^1^Molecular Microbiology and Bioenergetics, Institute of Molecular Biosciences, Johann Wolfgang Goethe-University of Frankfurt am MainFrankfurt am Main, Germany; ^2^Instituto de Tecnologia Química e Biológica, Universidade Nova de LisboaOeiras, Portugal

**Keywords:** *Halobacillus halophilus*, glutamine synthetase, compatible solutes, osmoregulation, halophile

## Abstract

*Halobacillus halophilus*, a moderately halophilic bacterium isolated from salt marshes, produces various compatible solutes to cope with osmotic stress. Glutamate and glutamine are dominant compatible solutes at mild salinities. Glutamine synthetase activity in cell suspensions of *Halobacillus halophilus* wild type was shown to be salt dependent and chloride modulated. A possible candidate to catalyze glutamine synthesis is glutamine synthetase A2, whose transcription is stimulated by chloride. To address the role of GlnA2 in the biosynthesis of the osmolytes glutamate and glutamine, a deletion mutant (Δ*glnA2*) was generated and characterized in detail. We compared the pool of compatible solutes and performed transcriptional analyses of the principal genes controlling the solute production in the wild type strain and the deletion mutant. These measurements did not confirm the hypothesized role of GlnA2 in the osmolyte production. Most likely the presence of another, yet to be identified enzyme has the main contribution in the measured activity in crude extracts and probably determines the total chloride-modulated profile. The role of GlnA2 remains to be elucidated.

## INTRODUCTION

Moderately halophilic bacteria are truly fascinating microorganisms that can grow over a wide range of salinities (from 0.5 to 3.0 M NaCl) with identical growth rates demonstrating a high flexibility in coping with salt stress. The molecular basis for this extraordinary capability of bacterial cells to adapt to these huge changes in salinity has been studied in several moderate halophiles such as the Gram negative *Halomonas elongata* (Vreeland et al., 1980; Cánovas et al., 1996, 1998; Ono et al., 1999; Grammann et al., 2002; Kurz et al., 2006) or the Gram positive *Halobacillus halophilus* (for a recent review, see Hänelt and Müller, 2013). The most prominent challenge a moderate halophile faces in its habitat is the loss of water from the cytoplasm at high salinities (Ventosa et al., 1998; Oren, 2008). This is combated by the accumulation of compatible solutes, small molecules that do not interfere with the primary metabolism (Galinski, 1995; Kempf and Bremer, 1998; Roeβler and Müller, 2001b; Roberts, 2005). *Halobacillus halophilus* accumulates glycine betaine, glutamate, glutamine, proline, and ectoine (Roeβler and Müller, 2001a; Müller and Saum, 2005; Saum et al., 2006; Saum and Müller, 2007, 2008a; Burkhardt et al., 2009). Most interestingly, *Halobacillus halophilus* switches its osmolyte strategy according to the salt concentration in the growth medium. Cells grown at moderate salinities (around 1 M NaCl) mainly synthesize glutamate and glutamine while at higher salinities (2.0–3.0 M NaCl) proline is the dominant compatible solute (Saum and Müller, 2007). In addition, *Halobacillus halophilus* not only accumulates compatible solutes but also chloride up to molar concentrations in the cytoplasm (Roeβler and Müller, 2002). Growth of *Halobacillus halophilus* is strictly dependent on the anion Cl^-^ (Roeβler and Müller, 1998). In line with this, various studies (Dohrmann and Müller, 1999; Roeβler and Müller, 2001a, 2002; Sewald et al., 2007; Köcher et al., 2009) unraveled a chloride modulon that mediates sensing of the external salt concentration and transmission of information to enzymes whose activities are modulated by chloride or to genes whose transcription is regulated by the anion (Saum and Müller, 2008b).

The routes for the biosynthesis of compatible solutes and their regulation in *Halobacillus halophilus* were examined in recent years. Biosynthesis of glutamate and glutamine occurs *via* glutamate dehydrogenase or the GOGAT cycle. The genome of *Halobacillus halophilus* contains two genes potentially encoding glutamate dehydrogenases and one encoding a glutamate synthase (Saum et al., 2012). Though their enzymes are probably involved in osmolyte production, transcriptional analysis did not reveal any effect of salt on their expression (Saum et al., 2006). Two genes potentially encoding glutamine synthetases, *glnA1* and *glnA2*, were identified in the genome of *Halobacillus halophilus*. Expression of *glnA2* but not *glnA1* increased up to 4-fold in cells adapted to high salt and was stimulated by chloride. Furthermore, glutamine synthetase activity increased with increasing salinities in the growth media in a chloride-dependent manner. These observations raised the hypothesis that GlnA2 is involved in the synthesis of the solutes glutamate and glutamine while the not upregulated GlnA1 most likely is part of the nitrogen metabolism (Saum et al., 2006). We decided to follow up the hints given by these observations and to address the role of GlnA2 in solute biosynthesis in *Halobacillus halophilus* using the recently established genetic system (Köcher et al., 2011). Consequently, the *glnA2* gene was deleted and the phenotype of the resulting mutant was characterized.

## MATERIALS AND METHODS

### ORGANISMS AND CULTIVATION

All strains used in this study are listed in **Table 1**. *Escherichia coli* DH5α was used as a general cloning strain (Hanahan, 1983) and grown under standard conditions (Ausubel et al., 1992). *Halobacillus halophilus* (DSMZ 2266) was routinely grown in glucose minimal medium (G10 medium) containing 50 mM glucose, 37 mM NH_4_Cl, 36 μM FeSO_4_ × 7 H_2_O, 100 mM Tris base, 3 mM K_2_HPO_4_, yeast extract (0.1 g·l^-1^), DSM 141 vitamin solution (1 ml·l^-1^), and DSM 79 artificial seawater (250 ml·l^-^^1^). The final concentration of NaCl varied depending on the assay conditions (values are given in the text). The pH was adjusted to 7.8 with H_2_SO_4_. *Halobacillus halophilus* was cultivated aerobically on a rotary shaker with 125 rpm at 30°C. Growth was monitored by measuring the optical density of the cultures at 578 nm (OD_578_). For protoplast transformation *Halobacillus halophilus* was grown in MB medium as specified by the manufacturer (Roth, Karlsruhe, Germany). For regeneration of protoplasts and selection of clones MB3 agar plates containing MB medium (Roth, Karlsruhe, Germany) supplemented with 0.5 M Na-succinate, 0.01% bovine serum albumin (BSA), 0.05% casamino acids (CAA), 0.5% glucose, and 0.8% agar were used. For selection of *Halobacillus halophilus* carrying the chloramphenicol acetyltransferase gene (*cat*) chloramphenicol was added from a sterile stock to a final concentration of 5 μg·ml^-^^1^.

**Table 1:** Strains used in this study.

Strain	Genotype	Reference
*Halobacillus halophilus* (DSMZ 2266)	Wild type	Claus et al. (1983)
*Halobacillus halophilus* Δ*glnA2*	Δ*glnA2*	This study
*Halobacillus halophilus* Δ*pro*	Δ*pro*	Köcher et al. (2011)
*Escherichia coli* DH5α (DSMZ 6893)	F-Φ80, *lacZ*ΔM15, *end*A1, *rec*A1, *hsd*R17 (r_k_^-^m_k_^+^), *sup*E44, *thi*-1, λ^-^, *gyr*A96, *rel*A1, Δ(lacZYA^-^*arg*F)U196	Hanahan (1983)

### CONSTRUCTION OF pHHΔ*glnA2*

**FIGURE 1:**
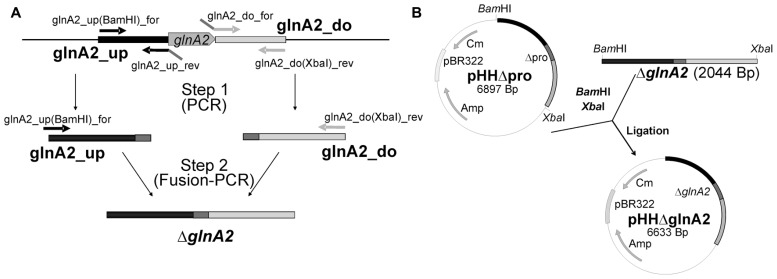
**Cellular concentration of GlnA1 and GlnA2 in dependence of different salinities in the growth media**. Cells of *Halobacillus halophilus* were cultivated in G10 minimal medium in the presence of varying NaCl concentrations (0.4–3 M). Samples were used to prepare cell-free extracts for SDS-Pages and Western blotting followed by densitometric analysis. All quantifications were carried out in duplicate using two independent cell cultures. A Coomassie blue-stained SDS-Page containing 20 μg protein per lane **(A)**. Western blot analysis using specific antibodies against GlnA1 **(B)** and GlnA2 **(C)** showing the cellular concentration of GlnA1 **(B)** and GlnA2 **(C)**, respectively, in dependence of salinities in the growth media. Averaged values of two independent Western blot analyses are given in the lower panel. The highest signal intensities were set to 100%.

Standard methods were used for construction of all plasmids. The primers and plasmids are listed in **Tables 2** and **3**, respectively. To construct a Δ*glnA2* DNA fragment, upstream and downstream regions of the gene were amplified using specific primers (*glnA2*_up_for, *glnA2*_up_rev and *glnA2*_do_for, *glnA2*_do_rev; **Table 2**). The downstream region of 1028 bp and the upstream region of 1044 bp were then fused together in a Fusion PCR (Dillon and Rosen, 1990). For this purpose, the 3′ end of the upstream fragment pro_up included a 26 bp overhang with homology to the 5′ end of the downstream fragment pro_do. A Fusion PCR with primers *glnA2*_up_for and *glnA2*_do_rev was performed as followed: one cycle at 94°C for 2 min, 25 cycles at 94°C for 30 s, 58°C for 1 min and 72°C for 2.5 min, followed by one cycle at 72°C for 10 min. The expected fragment Δ*glnA2* was purified from the gel using the High Pure PCR Product Purification Kit (Roche, Mannheim, Germany) and cloned into pHHΔ*pro* using the restriction enzymes *Bam*HI and *Xba*I (**Figure 1**). The construct with the size of 6633 bp was confirmed by sequencing. DNA sequences were retrieved from the genome sequence of *Halobacillus halophilus* (Saum et al., 2012).

**Table 2:** Oligonucleotides used in this study.

Name	Application	Sequence 5 ′ → 3^ ′
*glnA2*_up(BamHI)_for	Amplification of the region upstream *g1nA2* gene	CGGGATCCCGCATGTGCGTAGTTATGTACGTC
*glnA2*_up_rev	Amplification of the region upstream *glnA2* gene	CGATACAAATTCCTCGTAGGCGCTCGGGTATCTAAGTCTGGAACAAGG
*glnA2*_do_for	Amplification of the region downstream *glnA2* gene	CGAGCGCCTACGAGGAATTTGTATCGCACACAGCGCAGGCTTATATTG
*glnA2*_do(XbaI)_rev	Amplification of the region downstream *glnA2* gene	GATGACCCAGTCTCCTACGGGCTCTAGAGC
*glnA2*_downB	Verification of pHHΔ*glnA2* segregants	ATTGATACATGTTTCAGCACGATAGTAAAGAG
*glnA2*_upA	Verification of pHHΔ*glnA2* segregants	ATTATGCCGGCTATACAGTGGAGGACCCATCAA
*glnA2*_rev	Amplification of the *glnA2* probe (Southern blot)	TGGTTCTGCAGTTTTATTTGTTCATATATTTGTCG
*glnA2*_for	Amplification of the *glnA2* probe (Southern blot)	CGTAGTGCATATGACTTACACGAAAGAAACGATTAAGC

**Table 3:** Plasmids used in this study.

Plasmid	Description	Reference
pHHΔ*pro*	pTOPO derivative for generation of deletion mutants	Köcher et al. (2011)
pHHΔ*glnA2*	pTOPO derivative for generation of deletion mutants	This study

### PROTOPLAST TRANSFORMATION OF *Halobacillus halophilus*

The transformation procedure was performed as described before (Köcher et al., 2011). *Halobacillus halophilus* was grown in 200 ml MB medium in one liter Erlenmeyer flasks to the exponential growth phase (OD_578_ 0.6–0.8). After harvesting, the cells were washed in 2 ml SM3B buffer, which consists of 0.5 volume of twofold SMM buffer (1.0 M sucrose, 0.04 M maleate buffer, pH 6.5, and 0.04 M MgCl_2_) and 0.5 volume of twofold concentrated MB medium. The cell pellet was resuspended in 8 ml SM3B buffer containing 0.4 mg·ml^-1^ lysozyme. The suspension was incubated at 37°C with mild shaking for 1 h. Protoplast formation was monitored by phase-contrast microscopy. Protoplasts were harvested by centrifugation at 1,000 × *g* for 30 min. The precipitated protoplasts were resuspended in 1 ml SM3B and centrifuged again. The washed protoplasts were then resuspended in 500 μl SM3B and transferred to a 15-ml tube. 10–30 μg of pHHΔ*glnA2* were gently added to the protoplasts and mixed. 1.5 ml polyethylene glycol (PEG) 4000 (25% w/v, solved in 1× SMM) were added to the protoplast-DNA mixture, the suspensions were mixed and incubated at room temperature for 10 min. Subsequently, 5 ml SM3B were added and the protoplasts sedimented by centrifugation at 1,000 × *g* for 30 min. The precipitated protoplasts were resuspended in 2 ml SM3B buffer supplemented with 0.01% BSA and 0.05 μg·ml^-^^1^ chloramphenicol. For regeneration, the protoplasts were incubated aerobically at 30°C for 2 h before being plated on regeneration medium (MB3) agar plates containing 5 μg·ml^-^^1^ chloramphenicol. Regeneration plates were incubated at 30°C for 5–6 days. Due to the plasmid’s inability to replicate in *Halobacillus halophilus*, selection of the pHHΔ*glnA2* transformants for Cm^R^ resulted in *glnA2*/Δ*glnA2* merodiploid strains with the plasmid integrated into the chromosome *via* single homologous recombination. Resistant clones were isolated and the genotypes were verified by Southern blot analyses. For segregation, the strains were grown under non-selective conditions (MB medium, without chloramphenicol added) over 90 generations to allow resolution of the merodiploid state by recombination between the homologous sequences upstream and downstream of the *glnA2* gene. Different dilutions were plated on MB medium and single colonies were isolated and tested for Cm^S^. The genotypes of Cm^S^ clones obtained by screening *via* replica plating were verified by Southern blot analysis.

### SOUTHERN BLOT ANALYSIS

Sequence-specific probes were generated using “PCR DIG Labeling Mix” (Roche, Mannheim, Germany). Labeling reaction was performed according to the protocol supplied by the manufacturer. Two different probes were used for Southern blot hybridization, one against the flanking region of the mutated locus and one against the locus *glnA2*, which should be deleted. PCR primers that were used to generate labeled DNA fragments are listed in **Table 2**. Southern blot hybridization was performed as described by Ausubel et al. (1992) and signals were detected using the luminescent detection substrate CSPD as recommended by the manufacturer (Roche, Mannheim, Germany).

### DETERMINATION OF COMPATIBLE SOLUTES

Cells were grown in G10 medium to an OD_578_ of 0.3–0.6, harvested and freeze-dried. Secondary and tertiary amines were isolated and analyzed by HPLC as described previously (Kunte et al., 1993; Saum et al., 2006). Ectoine was quantified directly (Saum and Müller, 2008a). NMR analyses of compatible solutes with the same cells were carried out as previously described (Saum et al., 2009).

### SDS-PAGE AND IMMUNOBLOTS

Cells were resuspended in lysis buffer [50 mM Tris-HCl (pH 7.8), 20 mM NaCl, 0.1 mg·ml^-1^ lysozyme], incubated at 37°C for 15 min, and disrupted by sonication (four pulses; duty cycle: 50%; output control: 5) using a Branson Sonifier 250 (G. Heinemann Ultraschall- and Labortechnik, Schwäbisch Gmünd, Germany). Cell debris was separated by centrifugation for 10 min at 20,000 *g*. The supernatant was recovered and the protein content was determined using the assay described by Bradford (1976) with BSA as standard. 20 μg of protein was separated on a denaturing SDS gel (Laemmli, 1970) and blotted on a nitrocellulose membrane as described previously (Kyhse-Andersen, 1984). For detection, blot membranes were incubated in a mixture of 4 ml of solution A (200 ml containing 0.1 M Tris/HCl, pH 6.8, 50 mg luminol), 400 ml of solution B (10 ml dimethylsulfoxide containing 11 mg p-hydroxycoumaric acid) and 1.2 ml H_2_O_2_ for 2 min before exposure to WICORex film (Typon Imaging AG, Burgdorf, Switzerland).

### REAL-TIME PCR ANALYSIS

For real-time PCR analysis, *Halobacillus halophilus* cells were harvested in the early exponential growth phase (OD_578_ 0.15–0.3). RNA isolation and qPCR were done according to the standard protocol as described before (Saum et al., 2006). The primers used to amplify *glnA1, ectA*, and *proH* were published previously (Saum and Müller, 2007). Data analysis was accomplished applying the 2^-^^Δ^^Δ^^CT^ method (Livak and Schmittgen, 2001). Real-time PCR analysis was done with three independent physiological parallels to ensure statistical relevance. The open reading frame encoding malate dehydrogenase served as an internal normalizer. The expression of this gene did not change with changing salinities of the medium (Saum et al., 2006).

### ENZYMATIC ASSAY

Glutamine synthetase activity at whole cells was measured as described previously (Saum et al., 2006). For the preparation of cell suspensions, growth of cell cultures was stopped in the mid-exponential growth phase by adding 0.1% (w/v) cetyltrimethylammonium bromide (CTAB) and incubating the cells for 10 min on a shaker at 30°C. Cells were then harvested and washed in 0.2 volumes 1 M KCl corresponding to the NaCl concentration of 1 M in the growth medium. Finally, the pellet was resuspended in 1.5 M KCl to an OD_578_ of 70 and the cell suspension was stored on ice. The standard reaction mixture (4 ml) contained 126 mM imidazole hydrochloride, 17 mM hydroxylamine hydrochloride, 0.26 mM MnCl_2_, 24 mM potassium arsenate, 84 g·ml^-^^1^ CTAB, 0.37 mM Na-ADP, and varying final KCl concentration as indicated in the text. The pH was adjusted to 7.0. The cell suspension (0.5 ml) was preincubated with this mixture for 2 min at 37°C on a shaker, and the reaction was started by the addition of L-glutamine to a final concentration of 25 mM. Samples (0.5 ml) were withdrawn for 20 min in 5-min intervals, the reaction was stopped by the addition of 1 ml stop mix (0.2 M FeCl_3_, 0.15 M trichloroacetic acid, 0.25 M HCl), and the samples were incubated on ice for 30 min. Cells were removed by centrifugation (2 min, 15,000 × *g*). The formation of γ-glutamyl hydroxamate, which forms a brownish complex together with FeCl_3_, was measured at 540 nm. The purified enzyme was measured in forward reaction. The reaction mixture (800 μl) contained 94 mM Tris, 47 mM hydroxylamine hydrochloride, 56 mM MgCl_2_, 168 mM L-glutamate, and varying final KCl concentrations as indicated. The pH was adjusted to 7.0. The purified enzyme was preincubated for 5 min at 37°C with this mixture in a waterbath. The reaction was started by the addition of 10 mM ATP (final concentration). Samples (80 μl) were withdrawn for 10 min in 2.5 min intervals, and the reaction was stopped by the addition of 200 μl stop mix (0.2 M FeCl_3_, 0.15 M trichloracetic acid, 0.25 M HCl). The samples were incubated on ice for 30 min and precipitated protein removed by centrifugation (2 min, 15,000 × *g*). Formation of the γ-glutamyl-hydroxamate/FeCl_3_-complex was measured at 540 nm.

### PROTEIN CONTENT

80 μl perchloric acid (3 M) was added to 200 μl cell suspension. This mixture was incubated for 10 min at 100°C and then cooled on ice. After the addition of 1120 μl H_2_O and 466 μl trichloroacetic acid [25% (w/v)], precipitated protein was separated by centrifugation (15,000 × *g* for 15 min). The pellet was then resuspended in 400 μl Na_2_HPO_4_ buffer (20 mM) and 200 μl NaOH (0.1 M). The protein concentrations of these solutions as well as of cell extracts were determined by the method of Bradford (Bradford, 1976) using BSA as the standard. The protein content of CTAB permeabilized cells was determined before the addition of CTAB.

### PURIFICATION OF GlnA1 AND GlnA2 FROM *Halobacillus halophilus*

*Halobacillus halophilus* was grown in G10 medium with 1.5 M NaCl to the late-exponential growth phase. Cells were harvested *via* centrifugation and resuspended in buffer containing 25 mM Tris, 100 mM NaCl, 5 mM MgCl_2_, 10% glycerol, pH 8.0. After incubation with a final concentration of 0.1 mg·ml^-^^1^ lysozyme, cells were disrupted *via* french press in three passages at 1000 PSIG. Cellular debris was removed *via* centrifugation at 13,000 × *g* for 20 min. Cytoplasm and membranes were separated *via* ultracentrifugation at 48,000 × *g* for 1.5 h. The cytoplasm was removed carefully and submitted to a PEG-fractionation. First, 7% PEG_8000_ (final concentration) was added and the mixture stirred for 20 min. Precipitated protein was removed by centrifugation at 13,000 × *g* for 20 min. The glutamine synthetases of *Halobacillus halophilus* remained in the supernatant at this step. Protein pellet and supernatant were separated and the GlnA1- and GlnA2-containing supernatant was adjusted to 17% PEG_8000_ (final concentration). The mixture was again stirred for 20 min and the now precipitating glutamine synthetases pelleted *via* centrifugation (13,000 × *g* for 20 min). The GlnA1- and GlnA2-containing protein pellet was resuspended in fresh buffer (25 mM Tris, 100 mM NaCl, 5 mM MgCl_2_, 10% glycerol, pH 8.0) and the proteins were separated on an anion exchanger ResourceQ by a salt-gradient ranging from 100 to 550 mM NaCl. During this step, it was possible to separate GlnA1 and GlnA2, with GlnA1 eluting at low NaCl concentrations of about 220 mM, while GlnA2 eluted at higher NaCl concentrations of about 520 mM. Fractions that were shown to contain the corresponding glutamine synthetases by Western blot analysis and activity measurements were pooled and separated *via* Blue Sepharose column. Finally, each glutamine synthetase was separated from contaminating protein *via* gel filtration using a Superose 6 column.

## RESULTS

### CELLULAR CONCENTRATION OF GlnA2 IS DEPENDENT ON THE SALINITY

**FIGURE 2:**
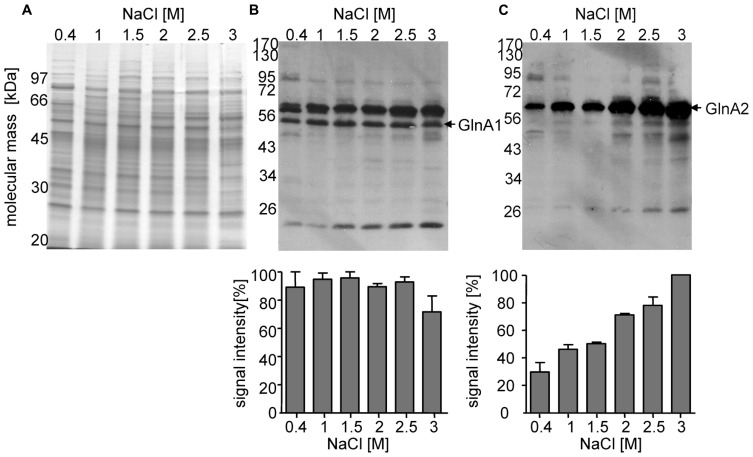
**Construction of pHHΔ*glnA2***. For construction of the non-replicating plasmid pHHΔ*glnA2* for the deletion of *glnA2* 1.0 kbp regions upstream and downstream of *glnA2* were amplified and merged by using fusion PCR to produce the fragment Δ*glnA2* **(A)**. After digestion with *Bam*HI and *Xba*HI the Δ*glnA2* fragment was cloned into the *Bam*HI and *Xba*HI sites of pHHΔ*pro* **(B)**.

In a previous study we found that the relative abundance of *glnA2* transcript increased at elevated salinities (Saum et al., 2006). Here, we analyzed the cellular abundance of GlnA1 and GlnA2 in relation to the external salinity. Cells were grown in G10 minimal media with NaCl concentrations ranging from 0.4 to 3 M, harvested, and disrupted by cell lysis and sonication. Consequently, 20 μg of protein from the cellular extract was separated by SDS-PAGE and the presence of GlnA1 and 2 were detected via Western Blot. With similar quantities of protein loaded per lane (**Figure 2A**), the amount of GlnA1 appeared to be consistent across all salinities tested (**Figure 2B**). GlnA2 appeared to increase with the salinity (**Figure 2C**). The threefold increase of the GlnA2 observed by increasing the salinity from 0.4 to 3 M NaCl is in good agreement with the fourfold increase in expression of *glnA2* gene determined previously (Saum et al., 2006) and supports the hypothesis that GlnA2 is the essential glutamine synthetase in solute production.

### CONSTRUCTION OF *Halobacillus halophilus* Δ*glnA2*

**FIGURE 3:**
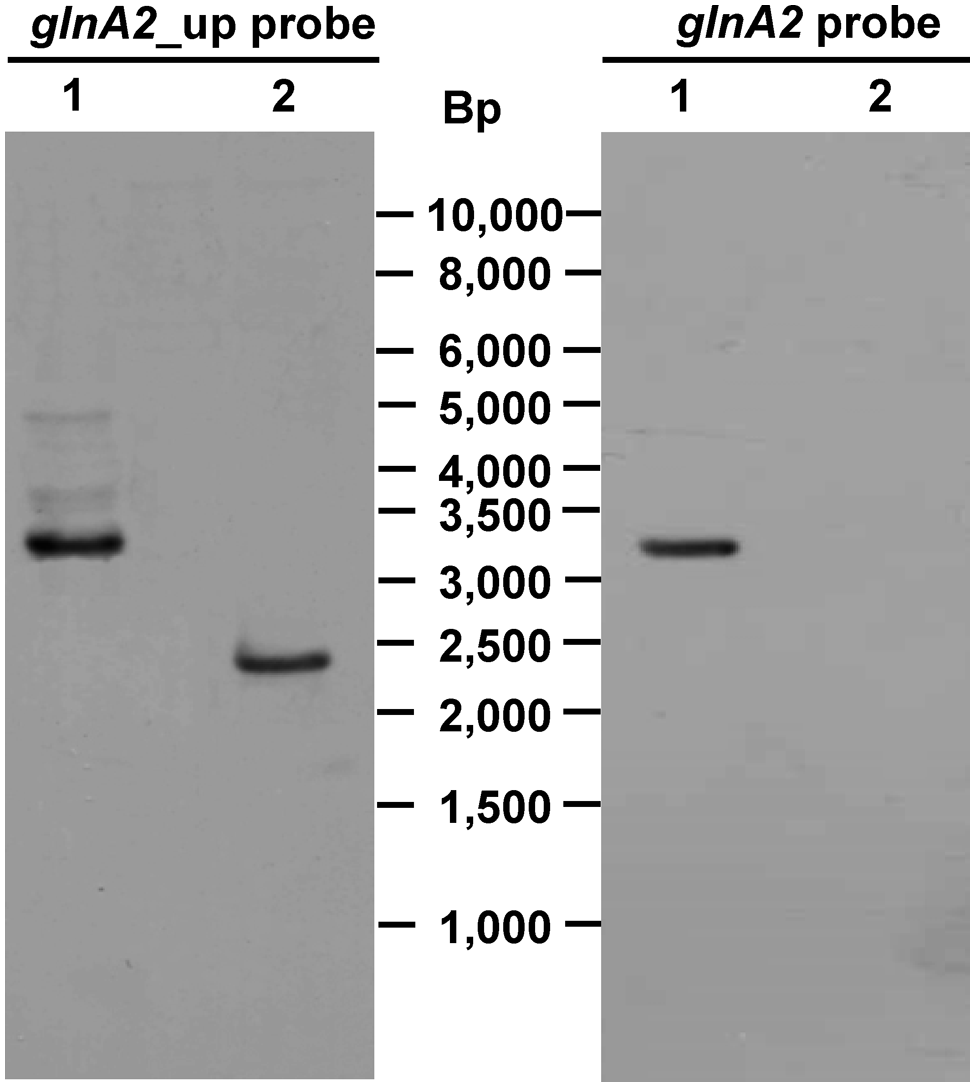
**Genotype of *Halobacillus halophilus* Δ*glnA2***. *Pst*I/*Bgl*I digested genomic DNA from *Halobacillus halophilus* wild type (lane 1) or *Halobacillus halophilus* Δ*glnA2* (lane 2) was separated by gel electrophoresis, transferred to a nylon membrane and probed with specific DIG-labeled DNA fragments against one flanking region of the mutated *glnA2* or the *glnA2* gene. Numbers in the middle indicate the migration positions of standard DNA fragments.

To delete the chromosomal copy of the *glnA2* gene, fragments of 1028 and 1044 bp upstream and downstream of *glnA2*, respectively, were amplified from the *Halobacillus halophilus* genomic DNA and fused together *via* fusion PCR to create the DNA fragment Δ*glnA2*. This fragment was then digested and cloned into pHHΔ*pro* resulting in the generation of pHHΔ*glnA2* (**Figure 1**). Aliquots of pHHΔ*glnA2* were used to transform *Halobacillus halophilus* by protoplast fusion. The transformation and subsequent segregation procedure was performed as described recently (Köcher et al., 2011). The genotype of the transformants was verified by Southern blot analysis (**Figure 3**). In the wild type the probe against the *glnA2* upstream region hybridized to a 3343 bp fragment, whereas the size of the complementary fragment was only 2444 bp in the mutant. The size of both fragments met the expectations from the calculated sizes (**Figure 3**, left panels). Using the probe against *glnA2*, no signal was detected in the Δ*glnA2* mutant, while a signal of the expected size (3343 bp) was detected in the wild type (**Figure 3**, right panels). These data demonstrate that pHHΔ*glnA2* had segregated from the chromosome and that the *glnA2* gene had been deleted. Similarly, we attempted to generate a Δ*glnA1* as well as a Δ*glnA1*Δ*glnA2* mutant. However, several attempts with varying conditions failed, which hints that the *glnA1* gene is essential.

### DELETION OF *glnA2* HAS NO INFLUENCE ON THE GROWTH OF *Halobacillus halophilus*

To test the effect of deleting *glnA2* on the cells’ ability to adapt to various salt concentrations, growth of the mutant was compared with that of the wild type in the presence of 1.0, 2.0, and 3.0 M NaCl dissolved in G10 medium. At all salinities tested, growth of *Halobacillus halophilus* Δ*glnA2* was similar to that of the wild-type strain in that the final optical densities and the growth rates were the same (data not shown). The fact that the loss of *glnA2* did not affect growth at different salt concentrations raised the question whether glutamate and glutamine are still accumulated to the same extent as in the wild type or whether other solutes compensate the deficiency of these osmolytes, especially at intermediate salinities (below 2 M NaCl).

### THE Δ*glnA2* DELETION HAS NO EFFECT ON THE COMPATIBLE SOLUTE POOL OF *Halobacillus halophilus* AT DIFFERENT SALINITIES

**FIGURE 4:**
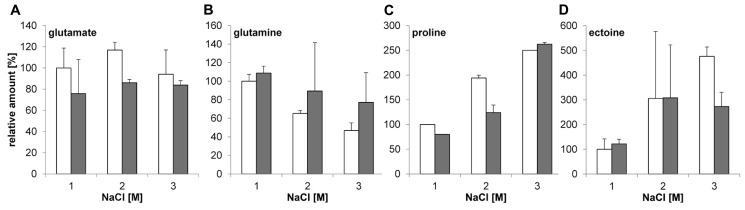
**Relative amounts of solutes in *Halobacillus halophilus* wild type and Δ*glnA2* mutant**. Cells were cultivated in G10 medium in the presence of 1.0, 2.0, or 3.0 M NaCl and harvested in the exponential growth phase. Compatible solutes were extracted and concentrations of glutamate **(A)**, glutamine **(B)**, proline **(C)**, and ectoine **(D)** were determined for wild type (white) and Δ*glnA2* mutant (gray) by HPLC. The presented relative quantification of solutes was conducted using the value of “wild type, 1.0 M NaCl” sample as a reference. The values represent the means and the standard deviations of the mean (S.D.s) of at least two physiologically independent parallels.

Since no growth phenotype was observed in the Δ*glnA2* mutant, the next goal was to test if there is any difference in the spectrum and the pool of compatible solutes in *Halobacillus halophilus* Δ*glnA2* compared to the wild type. Both strains were cultivated in G10 medium with 1.0, 2.0, and 3.0 M NaCl, respectively, to the exponential growth phase, harvested and lyophilized. The amount of glutamate, glutamine, proline, and ectoine was quantified by HPLC analyses as described previously (Saum et al., 2006). The results shown in **Figures 4A,B** clearly indicate that *Halobacillus halophilus*Δ*glnA2* is still able to produce glutamine and glutamate comparable to the wild type. Interestingly, production of glutamate and glutamine was not impaired indicating the presence of another enzyme responsible for their synthesis. With increasing salt concentrations proline became the main solute in the wild type, whereas the levels of glutamate and glutamine stayed constant (Saum and Müller, 2007). The same behavior was observed in *Halobacillus halophilus* Δ*glnA2.* Glutamate and glutamine concentrations in the wild type and in the Δ*glnA2* mutant stayed relatively constant over the whole range of salinities tested (**Figures 4A,B**). The proline content increased by a factor of 2.5 by elevating the salinity from 1 to 3 M NaCl in the wild type and by a factor of 2.63 in the Δ*glnA2* mutant at the same conditions (**Figure 4C**). As can be seen in **Figure 4D**, the intracellular ectoine concentration in the Δ*glnA2* mutant was similar to that of the wild type under identical conditions. The cellular level of ectoine increased by a factor of 4.75 in the wild type and by a factor of 2.73 in the Δ*glnA2* strain upon a salinity shift from 1 to 3 M NaCl. Thus, ectoine as well as proline accumulation were also not affected by the *glnA2* deletion. In addition, we addressed the question if the production of any alternative osmolytes is altered in *Halobacillus halophilus* Δ*glnA2* compared to the wild type. The wild type can synthesize minor amounts of alanine, *N*-acetyl lysine, and *N*-acetyl ornithine (Saum et al., 2009). To answer this question, NMR analysis of the same lyophilized cells used for HPLC analysis was conducted, but as for the most common solutes, no altered production of alternative solutes compared to the wild type was detected (data not shown).

### *glnA2* DELETION HAS NO EFFECT ON THE EXPRESSION LEVEL OF *glnA1*, *proH*, AND *ectA* IN *Halobacillus halophilus*

**FIGURE 5:**
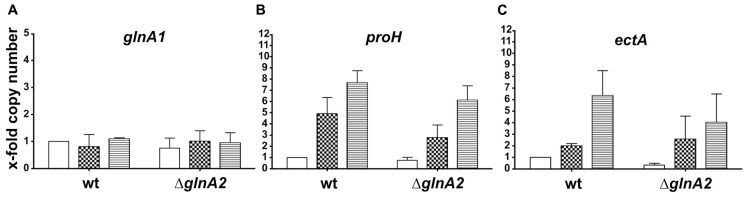
**Cellular transcript levels of *glnA1* (A), *proH* (B), and *ectA* (C) in *Halobacillus halophilus* wild type and Δ*glnA2* mutant**. Cellular transcript levels were detected in cells grown in G10 medium containing 1.0 (no fill), 2.0 (squares), or 3.0 (horizontal lines) M NaCl and harvested in the early exponential growth phase. The presented relative quantification of transcript levels was conducted using the value of “wild type, 1.0 M NaCl” sample as a reference. The experiment was repeated in two or three independent parallels to ensure statistical relevance.

Surprisingly, the glutamine and glutamate concentrations in *Halobacillus halophilus* were not affected by the deletion of *glnA2*. Therefore, we intended to identify the enzymes which keep the biosynthesis of these osmolytes at the same level as in the wild type. In order to determine if there is any impact of the loss of the *glnA2* gene on the expression of other genes responsible for the production of compatible solutes, relative transcription levels of the following genes were measured: *glnA1*, *proH* (the first gene of the *pro* operon), and *ectA* (the first gene of the *ect* operon). To quantify transcript levels of these genes in the Δ*glnA2* mutant, cells were grown in G10 medium in the presence of 1.0, 2.0, and 3.0 M NaCl, respectively, and harvested in the early exponential growth phase. Consequently, RNA was isolated, transcribed into cDNA and subjected to qRT-PCR. The levels of transcription in the wild type at 1 M NaCl were used as standard quantifiers for each gene and were set to 1. Since the most likely candidate to take over the function of glutamate and glutamine production in the Δ*glnA2* mutant is another glutamine synthetase, we expected an increase of the *glnA1* transcription level, especially at moderate salinities. In contrast, like in the wild type, constant *glnA1* expression levels were observed in this experiment (**Figure 5A**) regardless of the salinity in the growth medium. *glnA1* is not upregulated to complement the loss of GlnA2, but probably serves as the only glutamine synthetase crucial for glutamate and glutamine biosynthesis. Also for *ectA*, the first gene of the *ect* operon responsible for ectoine biosynthesis, the relative expression levels at all salinities tested were the same as in the wild type. Salinity-dependent dynamics did not change as a result of the deletion of *glnA2*. Due to an increase of the NaCl concentration from 1 to 3 M, the relative expression of *ectA* increased up to sevenfold in the wild type as well as in the Δ*glnA2* mutant (**Figure 5B**). The relative transcription level of *proH*, the first gene of the *pro* operon, was stimulated in the Δ*glnA2* strain up to eightfold by an increase of the salt concentration as it was observed in the wild type (**Figure 5C**). In conclusion, also a comparison of the relative transcriptional levels of *glnA1*, *proH*, and *ectA* in the Δ*glnA2* mutant and the wild type did not reveal any differences.

### GLUTAMINE SYNTHETASE ACTIVITY IN *Halobacillus halophilus* WILD TYPE AND IN THE Δ*glnA2* MUTANT IS SALT DEPENDENT BUT NEITHER GlnA2 NOR GlnA1 ARE CHLORIDE-MODULATED

**FIGURE 6:**
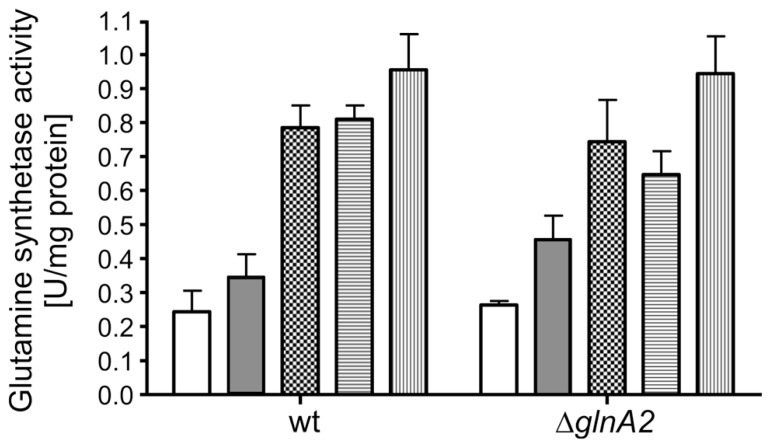
**Glutamine synthetase activity is salt-induced in *Halobacillus halophilus* wild type and Δ*glnA2* mutant**. Cells were grown in NB medium with 1 M NaCl, permeabilized and harvested in the late exponential growth phase. Cells were washed in an isoosmolar KCl solution, resuspended in 1.5 M KCl solution and exposed to the activity measurements, in which 0 (no fill), 0.5 (gray), 1.0 (squares), 2.0 (horizontal lines), and 3.0 (vertical lines) M KCl, respectively, were present. The values represent the means and the SEMs of three physiologically independent parallels.

**FIGURE 7:**
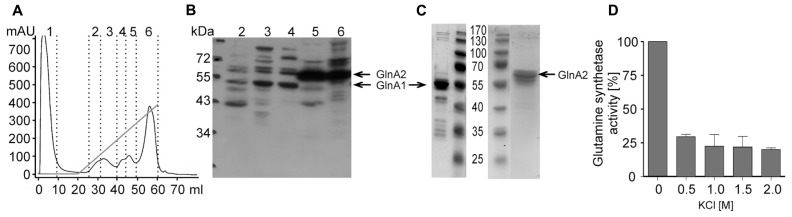
**Glutamine synthetase activities of purified GlnA1 and GlnA2**. *Halobacillus halophilus* was grown in G10 medium containing 1.5 M NaCl to the late exponential growth phase at 30°C. GlnA1 and GlnA2 were purified from the cytoplasm by two PEG precipitation steps followed by an anion exchanger. The chromatography profile **(A)** showed six major peak fractions, of which fractions 2–4 mainly contained GlnA1 while GlnA2 eluted in fractions 5 and 6 as shown by Western blotting **(B)**. Subsequently, GlnA1 and GlnA2 were enriched to higher purity by gelfiltrations of fraction 2–4 and 5–6, respectively **(C)**. Glutamine synthetase activities were measured for purified GlnA1 at different KCl concentrations in the assay **(D)**. GlnA2 has not shown any activity at the conditions tested.

In previous studies it was suggested that GlnA2 is responsible for the observed salt-dependent biosynthesis of glutamine and glutamate in cells since it was upregulated on a transcriptional level (Saum et al., 2006). However, as pointed out above, the accumulation of glutamate and glutamine was not affected in the Δ*glnA2* mutant at all salt concentrations tested. Consequently, one could hypothesize that possibly GlnA1 and not GlnA2 is the chloride-dependent glutamine synthetase in *Halobacillus halophilus* being involved in both, solute production and the nitrogen metabolism. To test this hypothesis we initially analyzed the glutamine synthetase activity at whole cells of both *Halobacillus halopilus* wild type and the Δ*glnA2* mutant. Cells were cultivated to the late-exponential growth phase in NB medium with 1 M NaCl. Activity measurements at whole cells were performed without salt or in the presence of 0.5, 1.0, 1.5, and 2.0 M KCl in the assay, respectively. In **Figure 6** the glutamine synthetase activities of the Δ*glnA2* mutant and of the wild type are demonstrated. The glutamine synthetase activity increased upon an increase of the KCl concentration in both strains and the activity values in the Δ*glnA2* mutant and in the wild type were very similar at the same conditions. Glutamine synthetase activity of the wild type which was 0.25 ± 0.1 U mg^-^^1^ protein without salt behaved in a salt-dependent manner and was upregulated to 0.95 ± 0.1 U mg^-^^1^ at 2 M KCl. The Δ*glnA2* mutant showed an increase of the glutamine synthetase activity from 0.25 ± 0.02 to 0.98 ± 0.15 U mg^-^^1^ upon the respective salinity increase. Apparently, the salt-stimulated enzyme was still present in the Δ*glnA2* mutant and thus the enzyme of choice was likely to be GlnA1. To further address that question on a molecular level, we decided to purify both GlnA1 and GlnA2 from crude cell extract to finally test the glutamine synthetase activity of the purified enzymes. For this purpose, *Halobacillus halophilus* cells were grown in G10 medium containing 1.5 M NaCl, harvested in the late-exponential growth phase and broken by three French press cycles. Subsequently, the broken cells were fractionated by a series of low speed and high speed centrifugation. The resulting cytoplasm contained both glutamine synthetases (data not shown). By two consecutive PEG precipitation steps, in which at the lower PEG concentration the glutamine synthetases remained in the supernatant while they precipitated at the increased PEG concentration, the enzymes were enriched. Finally, the proteins of the resuspended precipitate were separated *via* an anion exchanger by which also GlnA1 and GlnA2 were separated from each other due to their slightly different isoelectric points of 4.98 and 4.69, respectively (**Figures 7A,B**). The chromatography profile displayed six major peak fractions (**Figure 7A**), in which by Western blot analysis we identified GlnA1 mainly in fractions 2 to 4, while GlnA2 eluted later in fractions 5 and 6 (**Figure 7B**). To further purify GlnA1 and GlnA2, fractions 2–4 and 5–6, respectively, were pooled, concentrated and separated by gel filtration chromatography. By densitometry readings of SDS-PAGEs (**Figure 7C**), GlnA1 and GlnA2 were shown to be 62 and 75% pure, respectively. The enriched enzymes were analyzed for glutamine synthetase activity at varying KCl concentrations in the assay ranging from 0 to 2 M. In this assay GlnA2 showed no enzymatic activity at various conditions tested. In contrast, GlnA1 was active with a specific activity of 3.1 U mg^-^^1^. The highest activity was gained in absence of KCl while a reduction of 70% occurred in presence of 500 mM KCl already. A further increase of the salinity had no effect on the enzyme activity (**Figure 7D**). In summary, the deletion of *glnA2* had no effect on the glutamine synthetase activity in a whole-cell assay and at the same time, purified GlnA2 showed no activity at the conditions tested. This is in line with the observation that the deletion of *glnA2* has no effect on the glutamine and glutamate pools as well as with the finding that the loss of *glnA2* did not lead to the upregulation of the expression of the second gene encoding a glutamine synthetase, *glnA1*. GlnA1 instead showed the expected activity but as purified enzyme was not found to be salt or chloride dependent. Thus, GlnA1 most likely is the key enzyme for the synthesis of glutamine and glutamate in *Halobacillus halophilus*, but another, yet to be identified enzyme probably is responsible for the chloride-dependent activity as observed in whole-cell measurements.

## DISCUSSION

*Halobacillus halophilus* has two glutamine synthetases of the GSI type (Woods and Reid, 1993): GlnA1 and GlnA2. GlnA1 is encoded by *glnA1*, which is organized in an operon together with *glnR*. *glnR* most likely encodes a regulatory protein. This genetic organization is typical for *Bacillus sp.* and the derived amino acids sequence of *glnA1* is 81% identical to that of the corresponding protein from *Bacillus subtilis* (Saum et al., 2006), which is a classical enzyme involved in nitrogen metabolism. The second enzyme encoded by *glnA2* has a relatively unique amino acid sequence (52% identity to the glutamine synthetase from *B. subtilis*) and the respective gene lies solitary in the genome. Glutamate plays a crucial role in *Halobacillus halophilus* being involved in numerous metabolic and signaling pathways. Apart from serving as a structural unit in proteins, it can be used as single carbon and nitrogen source. It acts as a signaling molecule which triggers the production of compatible solutes proline and ectoine and can substitute chloride during cell growth (Saum and Müller, 2007). Furthermore, together with glutamine, it is the dominant compatible solute at moderate salt concentrations (below 2 M NaCl). Therefore, two homologs of GSI could possibly possess different functions; catalyze formation of glutamate and glutamine for different needs under specific conditions. Glutamine synthetase homologs in other organisms have diverse functions depending on their habitats and particular requirements. GlnA1 from *Mycobacterium tuberculosis* catalyzes the synthesis of L-glutamine, whereas GlnA2 is responsible for D-glutamine production and determines the virulence (Harth et al., 2005). Another glutamine synthetase homolog PA5508 produced in *Pseudomonas aeruginosa* is able to metabolize bulky amines rather than ammonia (Ladner et al., 2012). The nodulon/glutamine synthetase-like protein (NodGS) proteomically identified in *Arabidopsis thaliana* is a fusion protein containing a C-terminal domain of prokaryotic GSI. It does not possess glutamine synthetase activity, but plays a role in root morphogenesis and microbial elicitation (Doskocilova et al., 2011).

In this study, the role of GlnA1 and GlnA2 of *Halobacillus halophilus* was studied in detail by using a *glnA2* deletion strain and by analyzing the enzymatic activity of the purified enzymes. The growth phenotype of the Δ*glnA2* mutant, its accumulation of osmolytes, the expression of the genes encoding the key enzymes in osmolyte biosynthesis as well as its GS activity in comparison to the wild type were analyzed. Intriguingly, growth of the mutant at high salinities was not inhibited indicating that GlnA2 is not essential for optimal growth of *Halobacillus halophilus* at salt concentrations up to 3 M. Glutamate and glutamine pools were not altered as a result of the *glnA2* deletion. Since the proline biosynthesis occurs *via* glutamate, also its accumulation was investigated but no changes were detected. And finally, the possibility of secondary regulatory effects of the *glnA2* deletion on cellular concentrations of ectoine and some minor osmolytes such as *N*-acetyl-β-lysine, *N*-acetyl-ornithine, and alanine was verified. Ectoine was shown to be a minor solute at high salinities in *Halobacillus halophilus* wild type at the exponential growth phase (Saum and Müller, 2008a). But when the *proHJA* operon was deleted, the intracellular ectoine content increased up to 300% compared to the wild type (Köcher et al., 2011). However, no changes in these compatible solute pools in the Δ*glnA2* mutant compared to the wild type were observed. In accordance with pool sizes of compatible solutes, there was no influence of the *glnA2* deletion on the transcription of *proH* and *ectA*, whose gene products are responsible for the production of proline and ectoine, respectively, at different salinities. Upregulation of *glnA1* was not detected in the Δ*glnA2* mutant compared to the wild type. Taken together, there is actually no direct evidence for the role of GlnA2 in glutamate and glutamine production in *Halobacillus halophilus* in principle. The data obtained in this study clearly demonstrates that GlnA2 is not essential in *Halobacillus halophilus* and the function of the enzyme remains unknown. GlnA1 instead was shown to be active as purified enzyme and it is likely to be the only key enzyme in glutamate and glutamine production. If ever achieved, analysis of the phenotypes of a Δ*glnA1* and a double Δ*glnA1*Δ*glnA2* mutant might well clarify this question and is still considered as a next step in the investigation of glutamate and glutamine metabolism in *Halobacillus halophilus*. Deletion of *glnA* encoding a single glutamine synthetase in *E. coli* and *glnA* together with *gudB* in *B. subtilis* led to glutamine auxotrophy (MacNeil et al., 1982; Florez et al., 2011). A Δ*glnA* mutant of *Rhodopseudomonas capsulata* and a Δ*glnA1* mutant of *Rhodobacter sphaeroides* also showed the Gln^-^ phenotype, inability to assimilate ammonium and derepression of nitrogenase in presence of NH_4_^+^ (Scolnik et al., 1983; Li et al., 2010). Surprisingly, the *glnA* gene from *Rhodopseudomonas capsulata* could complement the Gln^-^ phenotype of an *E. coli glnA* deletion strain (Scolnik et al., 1983). Taken together, these data corroborate the conservative nature of *glnA* genes reported above and highlight the structural and functional similarities of glutamine synthetases within different classes of bacteria.

The Δ*glnA2* mutant and the wild type as well as the purified GlnA1 and GlnA2 proteins were additionally tested for salinity-dependent glutamine synthetase activity. Stimulation of GS activity in response to a salinity increase was clearly observed in both strains, the Δ*glnA2* mutant and the wild type. Besides, relative activity values in both strains were very similar under all conditions tested leading to the hypothesis that not GlnA2 but GlnA1 is the salt-regulated enzyme. Strikingly, neither purified GlnA1 nor GlnA2 showed a salt-stimulated activity. Therefore, we raised the question whether other chloride-induced enzymes are potentially co-measured by the applied methods. There are two common ways described in literature to measure potential glutamine synthetases: one is determining the formation of γ-glutamylhydroxamate from glutamate and the other the transfer from glutamine (Herzfeld, 1972). Though both methods can be used to analyze glutamine synthetases, they basically test different functions. While the first reports γ-glutamylhydroxamate synthetase activities (GS), the second determines L-glutamine-hydroxylamine glutamyltransferase activities (GT), which are not exclusively catalyzed by glutamine synthetases. The second method, which was used for whole-cell measurements in the present work as well as in previous studies in *Halobacillus halophilus*, thus primarily measures GT activity which is attributed mainly to GTs (Herzfeld, 1972; Herzfeld and Estes, 1973). Also proline-5-kinases, like ProJ involved in proline production in *Halobacillus halophilus*, catalyze GT activity. However, the deletion of *proJ* (Köcher et al., 2011) had no significant effect on GT activity in whole cells (data not shown). Therefore, the proline-5-kinase like both glutamine synthetases does not appear to significantly contribute the most to the chloride-dependent activity profile. The stimulation of the measured activity upon a salinity increase observed in the Δ*glnA2* mutant, in the Δ*pro* mutant as well as in the wild type is likely to be associated to GTs in *Halobacillus halophilus*, which still need to be identified.

## Conflict of Interest Statement

The authors declare that the research was conducted in the absence of any commercial or financial relationships that could be construed as a potential conflict of interest.
